# Effect of heterozygous pathogenic *COL4A3* or *COL4A4* variants on patients with X‐linked Alport syndrome

**DOI:** 10.1002/mgg3.647

**Published:** 2019-03-18

**Authors:** Yanqin Zhang, Jie Ding, Hongwen Zhang, Yong Yao, Huijie Xiao, Suxia Wang, Fang Wang

**Affiliations:** ^1^ Department of Pediatrics Peking University First Hospital Beijing China; ^2^ Department of Electron Microscopy Peking University First Hospital Beijing China

**Keywords:** Alport syndrome, genotype, heterozygous, phenotype, proteinuria

## Abstract

**Background:**

Alport syndrome is an inherited renal disease caused by mutations in *COL4A3*, *COL4A4,* or *COL4A5* genes. Coexisting mutations in either two of the three genes in Alport patients have been reported recently. However, the effect of heterozygous mutations in *COL4A3* or *COL4A4* genes in X‐linked Alport syndrome (XLAS) patients is unclear.

**Methods:**

Using targeted next‐generation sequencing, six unrelated Chinese children were identified to have a combination of a pathogenic variant in *COL4A5* and a heterozygous mutation in *COL4A3* or *COL4A4*. They were three males and three females. Another three XLAS males each with only one pathogenic variant in *COL4A5 *were included. The clinical data were analyzed and compared between the males in two groups (group 1, males with a pathogenic variant in *COL4A5 *and a heterozygous pathogenic variant in *COL4A3 *or *COL4A4*; group 2, males with only one pathogenic variant in *COL4A5*).

**Results:**

Patients with XLAS who also had heterozygous pathogenic *COL4A3 *or *COL4A4 *variants accounted for 1% of Alport syndrome. In this study, three children showed coexisting pathogenic variants in *COL4A5* and *COL4A3*. Two children showed pathogenic variants in *COL4A5* and *COL4A4*. One child had pathogenic variants in the three *COL4A3‐5* genes, in which the pathogenic variant in *COL4A5* was de novo and the pathogenic variants in *COL4A4* and *COL4A3* were inherited independently (in trans). The site and type of mutations in *COL4A5 *were similar between the two groups. It was revealed that males in group 1 presented more severe proteinuria than males in group 2 (*p < 0.05*).

**Conclusion:**

The present study provides further evidence for complicated genotype in Alport syndrome. For the first time, we reported a case with three pathogenic variants in *COL4A5*, *COL4A3*, and *COL4A4 *genes. Moreover, we found that heterozygous pathogenic *COL4A3* or *COL4A4* variants are likely to make XLAS disease more serious.

## 1 INTRODUCTION

Alport syndrome is a hereditary renal disease characterized by hematuria, proteinuria, progressive renal failure, and frequently with hearing loss or ocular abnormalities. The typical ultrastructural changes of kidney in Alport syndrome are diffuse glomerular basement membrane (GBM) lamellation (Flinter, 1997; Kashtan, 1999; Zhang & Ding, 2018). Alport syndrome is caused by mutations in the *COL4A3 *(OMIM, # 120070), *COL4A4* (OMIM, # 120131), or *COL4A5* (OMIM, # 303630) genes encoding collagen IVα3, α4, and α5 chains (Barker et al., 1990; Gross, 2008; Mochizuki et al., 1994). About 85% of individuals with Alport syndrome are X‐linked inherited due to mutations in the *COL4A5* gene. Males with X‐linked Alport syndrome (XLAS) are affected more severely than females (Massella et al., 2003; Raju, Cimbaluk, & Korbet, 2013; Savige, Colville, et al., 2016; Savige, Storey et al., 2016). Ninety percent of males with XLAS progress to end‐stage renal disease (ESRD) by age 40, but only about 20% of females with XLAS develop renal failure by age 60 (Jais et al., 2000, 2003; Naito, Kawai, Nomura, Sado, & Osawa, 1996; Wang, Ding, Guo, & Yang, 2002; Wang et al., 2012; Yamamura et al., 2017). Fifteen percent of individuals with Alport syndrome are autosomal recessive inherited caused by homozygous or compound heterozygous mutations from both alleles of either *COL4A3* or *COL4A4* genes (Storey, Savige, Sivakumar, Abbs, & Flinter, 2013; Wang et al., 2014; Zhang et al., 2012). Both males and females with autosomal recessive Alport syndrome (ARAS) have a high risk of ESRD by age of 30 (Kashtan et al., 2013; Oka et al., 2014).

However, the phenotype of individuals with heterozygous mutations in *COL4A3* or *COL4A4* genes varies widely. In our previous study, parents of ARAS children who were carriers of heterozygous mutations in *COL4A3* or *COL4A4* genes, 53% of them had normal urinalysis, 31% had hematuria, and 16% had hematuria and proteinuria (Zhang et al., 2012). The diseases associated with heterozygous mutations in *COL4A3* or *COL4A4* genes are thin basement membrane nephropathy and autosomal dominant Alport syndrome (Fallerini et al., 2014; Kamiyoshi et al., 2016; Savige et al., 2003).

To date, the effect of heterozygous mutations in *COL4A3* or *COL4A4* genes in XLAS patients is unclear. It is important to know whether it would make the XLAS disease worse. Here, we reported six unrelated Chinese children with XLAS who were also detected with heterozygous mutations in *COL4A3* or *COL4A4* genes. Our study aimed to provide more information on clinical assessment and genetic counseling for Alport syndrome.

## 2 MATERIALS AND METHODS

### 2.1 Patients and families

Patients diagnosed or suspected of Alport syndrome in the department of Pediatrics, Peking University First Hospital from 2014 to 2017 were screened for mutations in the *COL4A3*, *COL4A4*, *COL4A5,* and *COL4A6* genes. The clinical diagnosis criteria included ① glomerular hematuria, proteinuria, or renal failure, ② family history of Alport syndrome or renal failure without other exact disease, ③ lack or discontinuous staining of α5 (IV) chain in epidermal basement membrane (EBM), or in GBM, ④ the GBM lesions under electron microscopy (irregular thinning, thickening with splitting, and lamellation), ⑤ one pathogenic mutation in *COL4A5* or two pathogenic mutations in *COL4A3 *or *COL4A4 *genes. Alport syndrome was suspected for individuals with criteria ① and ②. Alport syndrome was diagnosed for individuals with criteria ① ② and one of ③ ④ ⑤.

Six unrelated XLAS children were found to have pathogenic variants in *COL4A5 *gene and also have heterozygous pathogenic variants in *COL4A3 *or *COL4A4* genes. They were three males and three females. Then another group of XLAS patients was enrolled in this study according to the following criteria: ① with only one pathogenic variant in *COL4A5* gene, ② with frameshift variant in exon 37, large deletion in exon 42, or glycine substitution in exon 25 of *COL4A5* gene, ③ male. Finally, there were three males of XLAS enrolled. Therefore, there were two groups of male patients (group 1, males with a pathogenic variant in *COL4A5 *and a heterozygous pathogenic variant in *COL4A3 *or *COL4A4*; group 2, males with only one pathogenic variant in *COL4A5*). For all subjects, clinical data including gender, age of disease onset, initial symptoms, extrarenal manifestations, results of skin biopsy and renal biopsy, family history, therapy, kidney function at latest follow‐up were collected.

### 2.2 Ethical compliance

The Ethical committee of Peking University First Hospital approved the project, and informed consent was obtained from the probands and their family members.

### 2.3 Genomic DNA and targeted NGS

A sample of peripheral blood in EDTA tubes was collected from probands and all available family members. Gnomic DNA was isolated from the blood samples using a FlexiGene DNA Kit (Qiagen) according to the manufacturer's protocol. Gnomic DNA from probands was detected for mutations in *COL4A3‐6* genes. Targeted next‐generation sequencing (NGS) was performed by BGI‐Tianjin, China as published previously (Wang et al., 2017). The pathogenicity of variants identified was based on meeting at least one of the following criteria: (a) truncating mutations (nonsense, consensus splice site ±1 or 2 nucleotide, large deletion, and frameshift), (b) variants previously described as disease causing in a patient with a similar phenotype in the website (HGMD, LOVD, and Clin Var), (c) Glycine missense variants in the intermediate collagenous domains (except p.Gly624Asp in *COL4A5*), or (d) novel non‐Glycine substitutions absent or at very low frequency in large population cohorts (1,000 genomes; ExAC, gnomAD), in domain high evolutionary conservation, and more than two prediction scores classified the allele as disease causing (SIFT, Mutation Taster, Polyphen 2) (Richards et al., 2015; Savige et al., 2018). The pathogenic mutations identified with NGS in probands were confirmed by Sanger sequencing or qPCR (real‐time quantitative PCR) for large deletion mutations. Gnomic DNA from family members was analyzed by Sanger sequencing or qPCR to determine whether they had the same mutations as the probands.

### 2.4 Statistical analysis

Statistical analysis was performed by SPSS 19.0. The differences of proteinuria level, age, and time of therapy in two groups were compared using unpaired *t *test. If the *p* value < 0.05, the differences were considered to be statistically significant.

## 3 RESULTS

In this study, among 417 patients diagnosed or suspected of Alport syndrome in our department during 2014–2017, six were identified with pathogenic variants in *COL4A5* plus heterozygous pathogenic variants in *COL4A3 *or *COL4A4*, which accounted for 1%. They were three males (proband 1, 2, 3) and three females (proband 4, 5, 6). Another three males (proband 7, 8, 9) with only one pathogenic variant in *COL4A5* were included. The sites and types of pathogenic variants in *COL4A5* gene were similar between the two groups of males. Two frameshift mutations in exon 37 p.G1110Afs*45 and p.G1098Vfs*54 in *COL4A5* gene were detected in proband 1 and proband 7, respectively. The same large deletion mutation (deletion of exon 42) in *COL4A5* gene was detected in both proband 2 and proband 8. Two missense mutations p.G594R and p.Gly644Val in exon 25 in *COL4A5* gene were detected in proband 3 and proband 9, respectively. The pathogenic variants identified in these children were shown in Table 1.

**Table 1:** Pathogenic variants in *COL4A3‐5 *genes identified in nine children with X‐linked Alport in this study

Proband number	Gender	Gene	Exon (intron)	Variant	Effect on protein	Type	dbSNP ID	SIFT (scroe)	PolyPhen2 (scroe)	Mutation Taster	Reference	Variant derived	
												Father	Mother
1	M	*COL4A5*	37	c.3328_3329insCAAACCAG	p.G1110Afs*45	Frameshift	—	—	—	DC	LOVD	—	Het
		*COL4A3*	47	c.4207G>A	p.G1403R	Missense	rs772528863	D(0)	PD(1)	DC	LOVD	—	N
2	M	*COL4A5*	42	Exon42 del	—	Exon deletion	—	—	—	—	—	—	—
		*COL4A3*	44	c.3946G>A	p.G1316S	Missense	—	D(0)	PD(1)	DC	LOVD	—	N
3	M	*COL4A5*	25	c.1780G>C	p.G594R	Missense	rs104886131	D(0)	PD(0.901)	DC	—	—	Het
		*COL4A4*	48	c.4915G>C	p.G1639R	Missense	rs749899964	D(0)	PD(1)	DC	LOVD	—	Het
4	F	*COL4A5*	1	c.50delT	p.L17Rfs*27	Frameshift	—	—	—	DC	—	N	N
		*COL4A3*	50	c.4664C>T	p.A1555V	Missense	rs369575989	D(0)	PD(1)	DC	—	N	Het
		*COL4A4*	32	c.2932G>A	p.G978R	Missense	rs759439914	D(0)	PD(1)	DC	—	Het	N
5	F	*COL4A5*	35	c.3094_3095delAT	p.M1032Gfs*35	Frameshift	—	—	—	DC	—	N	N
		*COL4A3*	32	c.2549G>A	p.G850E	Missense	—	D(0)	PD(1)	DC	—	Het	N
6	F	*COL4A5*	(47)	c.4511‐2A>G	—	Splicing	—	—	—	—	—	—	Het
		*COL4A4*	41	c.3826G>A	p.G1276R	Missense	—	D(0)	PD(1)	DC	—	—	Het
7	M	*COL4A5*	37	c.3293delG	p.G1098Vfs*54	Frameshift	—	—	—	DC	—	—	Het
8	M	*COL4A5*	42	Exon42 del	—	Exon deletion	—	—	—	—	—	—	—
9	M	*COL4A5*	25	c.1931G>T	p.Gly644Val	Missense	—	D(0)	PD(1)	DC	—	—	—

Note

Pathogenicity of missense variants predicted using SIFT, MutationTaster, and PolyPhen‐2.

The version number of COL4A3, COL4A4, or COL4A5 gene was NM_000091.4, NM_000092.4, and NM_033380.2, respectively.

D: deleterious; PD: probably damaging; DC: disease causing; N: normal; Het: heterozygous; LOVD: Leiden open variation database.

The clinical manifestations were compared between the two groups of XLAS males (Table 2). All the boys in the two groups had initial symptoms of hematuria and proteinuria, and positive family history of kidney disease. The onset age of the disease, and hearing loss rate and age had no difference between the two groups. Kidney biopsy information was available in four boys. Skin biopsy and α5 (IV) chain staining in the EBM were underwent in three boys and two of their mothers. Negative staining of collagen IV α5 chain was identified in either EBM or GBM in five boys. All the six boys had angiotensin‐converting enzyme inhibitor (ACEI) and angiotensin receptor blocker (ARB) for treatment after diagnosis. The amount of ACEI and ARB for each patient was 0.25 mg^−1^kg^−1^d and 0.5 mg^−1^kg^−1^d, respectively. The length of time for therapy and the age at latest follow‐up had no difference between the two groups. All the boys had normal renal function at latest follow‐up. However, the proteinuria during follow‐up was more severe in three boys (proband 1, 2, 3) with a combination of one pathogenic variant in *COL4A5* and one heterozygous pathogenic variant in *COL4A3* or *COL4A4.* And the difference between the two groups was statistically significant (*p < 0.05*).

**Table 2:** Comparison of clinical manifestations of two groups of males with X‐linked Alport syndrome

Proband number	Sex	Onset age (years)	Initial symptom	Family history of kidney diseases	Extrarenal manifestations (age, years)	α5 (IV) chain staining in the EBM	Renal biopsy(age, years)		Treatment (onset age, years)	Latest follow‐up				
							Electron microscope findings	α5 (IV) chain staining		Age (years)	Urinary RBC/HP	24‐hr urinary protein (g/d)	Ccr(ml/1.73m^2^/min)	Scr (umol/L)
1	M	11.8	Hematuria, proteinuria	Yes	Hearing loss(6)	ND	Thinning, thickening and splitting, basket‐weaving of GBM(11.8)	Negative staining of α5 (IV) chain in GBM, BC, TBM	ACEI and ARB (11.8)	11.8	15	3.08	132.6	36.3
2	M	12	Hematuria, proteinuria	Yes	Hearing loss(14)	ND	ND	Negative staining of α5 (IV) chain in GBM, BC, TBM (13)	ACEI and ARB (12)	14	5	3.65	189.5	68
3	M	5	Hematuria, proteinuria	Yes	ND	Negative; discontinuous staining (the mother)	ND	ND	ACEI and ARB (5)	16	ND	1.63	145.21	65.9
7	M	11.3	Hematuria, proteinuria	Yes	Hearing loss(11)	ND	ND	ND	ACEI and ARB (11)	11	40–50	0.59	116.41	54.5
8	M	2.3	Hematuria, proteinuria	Yes	Hearing loss(12)	Negative; discontinuous staining (the mother)	Thinning of GBM(2)	Negative staining of α5 (IV) chain in GBM, BC, TBM	ACEI and ARB (3)	13	50–100	0.47	97.24	59.5
9	M	9	Hematuria, proteinuria	Yes	Hearing loss(9)	Negative	Thinning, thickening and splitting of GBM (14)	ND	ACEI and ARB (14)	14	20–30	1.17	138	74

Note

Group 1: proband 1, 2, 3 (males with one pathogenic variants in *COL4A5* and one heterozygous pathogenic variant in either *COL4A3* or *COL4A4*); Group 2: proband 7, 8, 9 (males with only one pathogenic variant in *COL4A5*).

EBM: epidermal basement membrane; GBM: glomerular basement membrane; BC: Bowman's capsule; TBM: tubular basement membrane; ACEI: angiotensin‐converting enzyme inhibitor; ARB: angiotensin receptor blocker; α5 (IV): collagen IV α5 chain; Ccr: creatinine clearance rate; Scr: serum creatinine concentration; ND: no data.

In the three females (proband 4, 5, 6), the initial symptom was hematuria and the onset age was from 5 months to 3.6 years (Table 3). During follow‐up, proteinuria was presented in proband 6 at 5 years and was 0.207 g/d at 8 years under ACE inhibitor therapy. Proband 4 was a 3.6‐year‐old girl with three pathogenic variants in *COL4A5*, *COL4A3*, and *COL4A4*. She had no family history. Hearing was not detected. Renal biopsy at 3.6 years showed thinning and thickening of GBM. She was presented with hematuria and proteinuria at last follow‐up (3.8 years).

**Table 3:** Clinical manifestations of three females with complicated genotype

Proband number	Sex	Onset age (years)	Initial symptom	Family history of kidney diseases	Extrarenal manifestations (age, years)	α5 (IV) chain staining in EBM	Renal biopsy (age, years)	Treatment (onset age, years)	Latest follow‐up				
									Age (years)	Urinary RBC/HP	24‐hr urinary protein (g/d)	Ccr (ml/1.73m^2^/min)	Scr (Umol/L)
4	F	3.6	Hematuria	No	ND	ND	Thinning and splitting of GBM (3.6)	No	3.8	100–120	a	NA	NA
5	F	5 months	Hematuria	Yes	ND	ND	ND	No	4	ND	0.12	NA	33.9
6	F	2	Hematuria	Yes	ND	ND	Thinning and thickening of GBM (4)	ACEI(5)	8	Full visual field	0.207	NA	33

Note

EBM: epidermal basement membrane; GBM: glomerular basement membrane; ACEI: angiotensin‐converting enzyme inhibitor; Ccr: creatinine clearance rate; Scr: serum creatinine concentration; ND: no data.

a urinary protein creatinine ratio 0.32 g/g.

**Figure 1:**
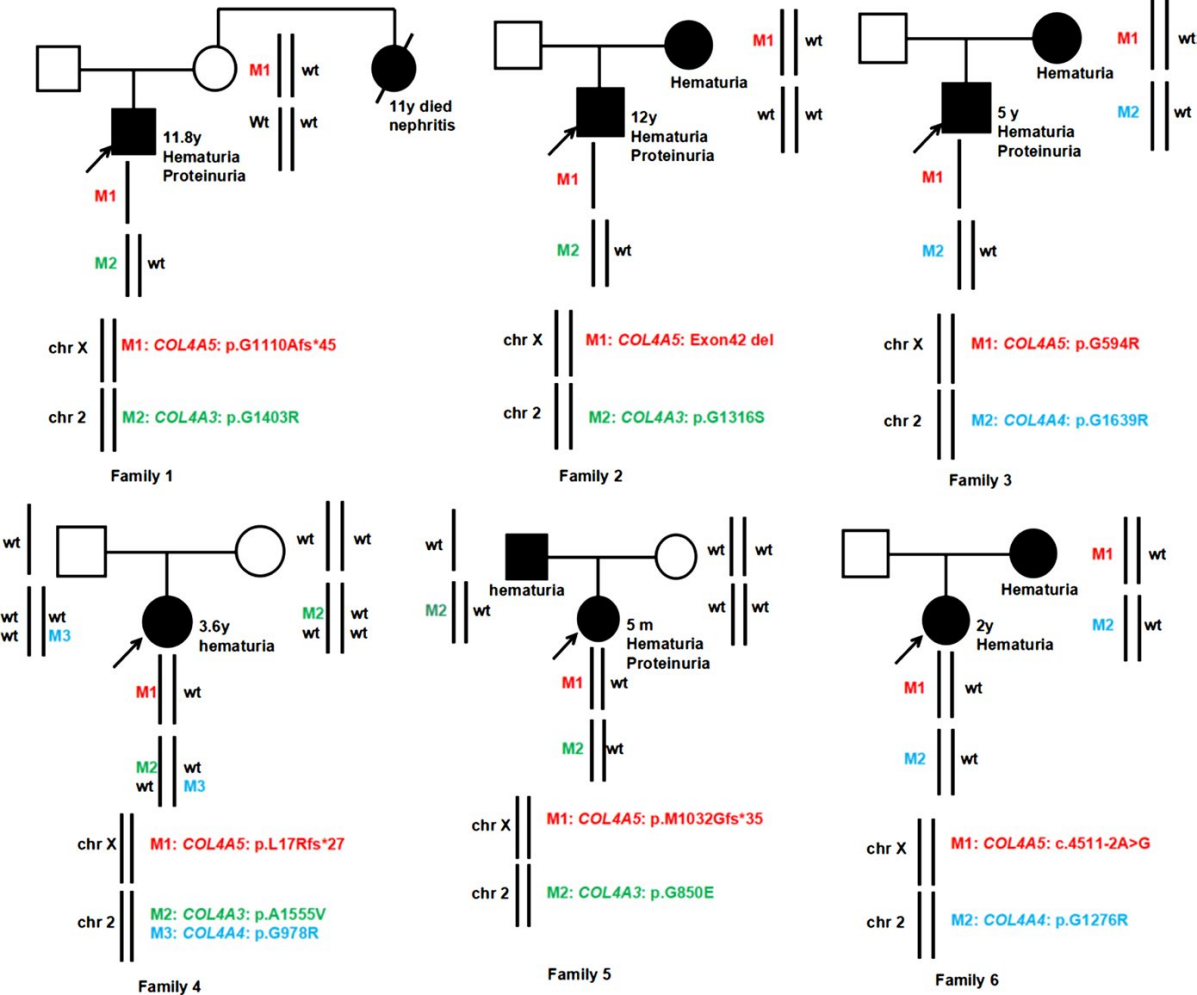
Pedigrees of six families presenting with mutations in more than one of three *COL4A3‐5* genes. Proband is indicated by an arrow. Square indicates male; circle, female; black symbol, individual with clinical symptoms

Family segregation analysis was performed in all available family members. In proband 1 and proband 2, only their mothers’ samples were available and it revealed the pathogenic variants in *COL4A5 *were inherited from the mother, but the pathogenic variants in *COL4A3 *were not from the mother. In proband 3 and proband 6, it revealed that both of the pathogenic variants in *COL4A5* and *COL4A4* genes were inherited from the mother. And the pathogenic variants in *COL4A5* gene were de novo in proband 4 and proband 5. In addition, the two heterozygous pathogenic variants in *COL4A3* and *COL4A4* genes in proband 4 were inherited in trans (on opposite chromosomes) from the mother and the father, respectively. The pedigrees of the six families were shown in Figure 1.

## 4 DISCUSSION

The present study provides further evidence for complicated genotype in Alport syndrome. About 1% of patients diagnosed or suspected for Alport syndrome had mutations in more than one of the *COL4A3‐5 *genes. For the first time, we reported a case with three pathogenic variants in *COL4A5*, *COL4A3*, and *COL4A4 *genes. Moreover, we found initially XLAS males with an additional heterozygous pathogenic *COL4A3 *or *COL4A4 *variant presented heavier proteinuria than the XLAS males with only one pathogenic variant in *COL4A5*.

So far, only two males with pathogenic variants in *COL4A5 *and *COL4A4* genes have been reported (Fallerini et al., 2017; Mencarelli et al., 2015). One was 26 years old with variant p.Gly1348Arg in *COL4A5 *and heterozygous variant p.Gly722Ser in *COL4A4* presented with hematuria and proteinuria. Another male with variant p.Gly684Val in *COL4A5 *and heterozygous variant p.Pro1587Arg in *COL4A4 *had ESRD. The onset age of ESRD was not recorded. In addition, three females with combination of a pathogenic variant in *COL4A5* and a heterozygous pathogenic variant in *COL4A3* or *COL4A4* had been reported. Their age was 9, 45, and 54 years old. The 9‐year‐old female was presented with hematuria and proteinuria. The 45‐year‐old female was presented with hematuria, proteinuria, and hearing loss. The 54‐year‐old female had ESRD at the age of 44. Therefore, these reported data from adult patients were consistent with our results that an additional heterozygous pathogenic *COL4A3 *or *COL4A4 *variant would make XLAS disease worse.

There were patients reported to have a combination of pathogenic variants in *COL4A3* and *COL4A4 *(Fallerini et al., 2017; Kashtan et al., 2018; Mencarelli et al., 2015)*. *In some cases the variants were on the same homologous chromosome (in cis). But in other cases the variants were inherited independently (in trans), like proband 4 in our study. Learning from the reported data, individuals with two heterozygous pathogenic variants in *COL4A3* and *COL4A4* in trans had more severe phenotype than those with a single heterozygous pathogenic variants, but had less severe phenotype than ARAS. However, there was no reference about XLAS patients combined with two heterozygous pathogenic variants in *COL4A3* and *COL4A4* in trans. The proband 4, a 3.6‐year‐old girl, reported here is the first case with this kind of complicated genotype. It is unknown whether her phenotype would be more severe than females with XLAS or ARAS. The long‐term follow‐up and more cases in the future might let us know more.

Besides, we should be alert that the three XLAS males with heterozygous pathogenic *COL4A3 *or *COL4A4 *variant in this study showed negative staining of α5 (IV) chains in EBM or GBM. A clinical diagnose of XLAS is definite (Hashimura et al., 2014; Savige et al., 2013; Wang et al., 2012; Wei et al., 2006). However, the genotype may be various, which would affect the risk of inheritance in families (Artuso et al., 2012; Kashtan et al., 2018). Therefore it is necessary to test all the three genes *COL4A3‐5 *by NGS in patients suspected for Alport syndrome, not only meaningful for diagnosis, but also for the genetic counseling (Gross et al., 2017; Kashtan et al., 2018; Savige, Colville, et al., 2016).

In conclusion, we reported six XLAS children with heterozygous pathogenic *COL4A3 *or *COL4A4 *variants, which accounted for 1% of Alport syndrome. One of them was the first case with three pathogenic variants in *COL4A5*, *COL4A3*, and *COL4A4 *genes. Our data revealed an additional heterozygous pathogenic *COL4A3 *or *COL4A4 *variant would make XLAS males suffering from more severe proteinuria. It suggested that another genetic hit from *COL4A3 *or *COL4A4 *might make the XLAS disease worse.

## CONFLICT OF INTEREST

The authors have no conflict of interest to declare.
